# Food marketing in recreational sport settings in Canada: a cross-sectional audit in different policy environments using the Food and beverage Marketing Assessment Tool for Settings (FoodMATS)

**DOI:** 10.1186/s12966-018-0673-5

**Published:** 2018-05-31

**Authors:** Rachel J. L. Prowse, Patti-Jean Naylor, Dana Lee Olstad, Valerie Carson, Kate Storey, Louise C. Mâsse, Sara F. L. Kirk, Kim D. Raine

**Affiliations:** 1 School of Public Health, University of Alberta, Edmonton, Alberta Canada; 2 School of Exercise Science, Physical and Health Education, University of Victoria, Victoria, British Columbia Canada; 3 Department of Community Health Sciences, Cumming School of Medicine, University of Calgary, Calgary, Alberta Canada; 4 Faculty of Physical Education and Recreation, University of Alberta, Edmonton, Alberta Canada; 5 BC Children’s Hospital Research Institute, Vancouver, British Columbia Canada; 6 School of Population and Public Health, University of British Columbia, Vancouver, British Columbia Canada; 7 Healthy Populations Institute, Dalhousie University, Halifax, Nova Scotia Canada; 8 Centre for Health and Nutrition, University of Alberta, Edmonton, Alberta Canada

**Keywords:** Food marketing, Recreational sport settings, Children and youth, Policy, Healthy eating

## Abstract

**Background:**

Children’s recreational sport settings typically sell energy dense, low nutrient products; however, it is unknown whether the same types of food and beverages are also marketed in these settings. Understanding food marketing in sports settings is important because the food industry often uses the promotion of physical activity to justify their products. This study aimed to document the ‘exposure’ and ‘power’ of food marketing present in public recreation facilities in Canada and assess differences between provinces with and without voluntary provincial nutrition guidelines for recreation facilities.

**Methods:**

Food marketing was measured in 51 sites using the Food and beverage Marketing Assessment Tool for Settings (FoodMATS). The frequency and repetition (‘exposure’) of food marketing and the presence of select marketing techniques, including child-targeted, sports-related, size, and healthfulness (‘power’), were assessed. Differences in ‘exposure’ and ‘power’ characteristics between sites in three guideline provinces (*n* = 34) and a non-guideline province (*n* = 17) were assessed using Pearson’s Chi squared tests of homogeneity and Mann-Whitney U tests.

**Results:**

Ninety-eight percent of sites had food marketing present. The frequency of food marketing per site did not differ between guideline and non-guideline provinces (media*n* = 29; *p* = 0.576). Sites from guideline provinces had a significantly lower proportion of food marketing occasions that were “Least Healthy” (47.9%) than sites from the non-guideline province (73.5%; *p* < 0.001). Use of child-targeted and sports-related food marketing techniques was significantly higher in sites from guideline provinces (9.5% and 10.9%, respectively), than in the non-guideline province (1.9% and 4.5% respectively; *p* values < 0.001). It was more common in the non-guideline province to use child-targeted and sports-related techniques to promote “Least Healthy” items (100.0% and 68.4%, respectively), compared to the guideline provinces (59.3% and 52.0%, respectively).

**Conclusions:**

Recreation facilities are a source of children’s exposure to unhealthy food marketing. Having voluntary provincial nutrition guidelines that recommend provision of healthier foods was not related to the frequency of food marketing in recreation facilities but was associated with less frequent marketing of unhealthy foods. Policy makers should provide explicit food marketing regulations that complement provincial nutrition guidelines to fulfill their ethical responsibility to protect children and the settings where children spend time.

## Background

Increased prevalence of childhood obesity is believed to be the product of “small, cumulative environmental changes that have altered children’s physical activity and dietary patterns” (p.e1) [1]. By providing opportunities to be active, recreation and sport facilities may be ideal sites to support childhood obesity prevention. Recreation and sport facilities, defined as public or private community centres that offer opportunities for physical activity and programming for children and adults at a fee, have a mandate to promote health and wellbeing [2]. However, this mandate may be undermined by the unhealthy foods they offer [3] which are commonly deep fried foods, hot dogs, and sugary snacks and drinks [4]. In a systematic review by Nelson et al. [5], no difference in children’s weights was found between those who participated in extracurricular physical activity and those who did not, in spite of the former being more physically active than the latter. Increased availability, marketing and consumption of fast foods and soft drinks in sport settings may have contributed to this weight discrepancy [5].

Food and beverage marketing (henceforth food marketing) in recreation and sport facilities may influence food attitudes, preferences and behaviors. A scoping review of the relationship between watching sports and population health concluded that sport spectating may increase unhealthy eating behaviours from exposure to unhealthy food sponsorship [6]. Unhealthy food marketing that uses sport or physical activity appeals is concerning due to its associated impacts on product likeability and nutritional quality. In a cross-sectional study of 10–14 year olds who participated in sports at a local club in Australia, over two-thirds could recall at least one food and beverage company sponsor of their club and 59% “liked to return the favour to these sponsors by buying their products” (p.4) [7]. Furthermore, both adults and children may experience a ‘halo effect’ when food is marketed with physical activity themes, leading to more positive reactions and perceptions of product healthfulness [8].

Restricting unhealthy food and beverage sport sponsorship and improving healthy food availability in recreation and sport facilities have been ranked as some of the most important and feasible interventions to promote children’s health [9]. In this regard, several Canadian provinces [Alberta (AB), British Columbia (BC), Nova Scotia (NS)] have introduced voluntary nutrition guidelines to encourage healthier food provision in recreation facilities [10–12]. Guidelines introduced in 2015 in NS, Canada discouraged unhealthy food promotion, sponsorship, and marketing [12]. Taking a different approach, guidelines in AB, Canada, revised in 2012, recommended marketing healthier foods through competitive pricing and placement [11]. Guidelines in the Canadian province of BC, revised in 2014, did not mention food marketing [10]. Even without specific food marketing recommendations, food marketing environments may improve in parallel with improved food provision as guidelines are implemented in recreation facilities. Once a new food product introduced into a recreation facility, marketing may be used to increase consumers’ “recognition, appeal and/or consumption” [13] (p.9) of the product through pricing, placement, or promotion [14]. Thus, we aimed to investigate the difference in food marketing environments between provinces with and without provincial nutrition guidelines.

Describing the nature and extent of food marketing in sport settings is a current gap in the literature [6]. The limited available research focuses on the prevalence of sport sponsorship [15] or testing the impact of experimental food marketing techniques in recreation facilities on food choices [16, 17]. It is necessary to understand the breadth, intensity, and characteristics of food marketing in recreation facilities to inform healthy food policy and reduce children’s exposure to unhealthy food marketing. Marketing policies that reduce ‘exposure’ to and ‘power’ of food and beverage marketing are recommended by the WHO [18] and could reduce the impact of unhealthy food marketing on children’s eating behaviors.

To fill the gap in the literature regarding food marketing in recreation facilities, this study aimed to document the food and beverage marketing in public recreation and sport facilities in Canada and assess differences in food marketing environments between facilities from provinces with voluntary nutrition guidelines and facilities from a province with no guidelines. This type of investigation is valuable as it may reveal how well current nutrition guidelines designed to enhance healthy food provision also protect (or do not protect) children from unhealthy food marketing. We aimed to explore the ‘exposure’ (frequency, repetition) to and ‘power’ (healthfulness, child-targeting, sports-related, size) of food marketing in public recreation facilities. We hypothesized that recreation facilities in provinces with voluntary nutrition guidelines would have less unhealthy food marketing (related to a difference in food provision) but did not have any other a priori hypotheses for other outcomes assessed due to limited research that currently exists on this topic.

## Methods

### Setting and participants

This study was part of a larger *Eat Play Live* (EPL) research project evaluating the impact of voluntary provincial nutrition guidelines on recreation and sport facility food environments including food availability, marketing, and policy in Canada. Public recreation facilities in three provinces with existing provincial nutrition guidelines for recreation facilities (BC [10], AB [11], and NS [12]) and one province without provincial nutrition guidelines [Ontario (ON)] were included in the current study. Eligible facilities were those that provided food services through vending or concession (such as a canteen, snack bar, café, or restaurant), had not made major changes to their food environment since 2010, were willing and able to make changes to their food environment, and had year-round sport programming.

Facilities were recruited for EPL between August 2015 and April 2016 by provincial parks and recreation organizations and the EPL team. A buffer of 150 km (adjusted by provinces if appropriate in regards to geography and budget) was used to identify a subsample of facilities near universities (*n* = 286) that were followed-up by telephone. Only 216 facilities were eligible to participate and 49 facilities (22.7%) agreed to participate. Of the remaining, 141 did not respond to the invitation; 11 refused without reason; 15 refused due to insufficient staff capacity (*n* = 11), uninterested in research (*n* = 2), risk of being a control site (*n* = 1), worried about competition (*n* = 1). Non-response greatly varied by province (ON 25%; BC 36%; AB 63%; NS 92%). Two facilities had two separate buildings which we treated as individual sites for a total of 51 sites where food and beverage marketing was measured. Thirty-four sites were from the three guideline provinces; 17 sites were from the one non-guideline province. A sample size of 43 was required for the EPL project to detect a medium to large effect (d = 0.8) in unhealthy food and beverage availability in vending machines between two groups with α =0.05. See methods for post hoc power analyses of the sample size to detect change in marketing scores.

### Data collection

A trained EPL provincial coordinator or research assistant conducted observational audits using the Food Marketing Assessment Tool for Settings (FoodMATS) [19] between November 2015 and May 2016. The FoodMATS captures the presence of food marketing in recreation facilities, what food products, brands, and retailers were marketed, and whether persuasive (powerful) marketing techniques were used. At each site, a trained rater photographed and recorded the following on the FoodMATS:

- the frequency of food and beverage marketing in sports areas, food areas (concessions), and other areas (entrance, hallways, parking lot),
- the product, brand, or food retailer marketed,
- whether the marketing occasion targeted children,
- whether the marketing occasion was related to sports, and
- the physical size of the marketing occasion.

One marketing occasion was defined as one advertisement, promotion, or message (e.g. one sign), that is intended to increase the “recognition, appeal and/or consumption” of a food or beverage products, brands, or retailer [13] (p.9). Marketing occasions that were not physical signage (e.g. product placement and pricing promotions) were counted but were not assessed for targeting children, being related to sports, or their size as that would usually require reviewing product packaging which was beyond the scope of this study.

After each site visit, one registered dietitian (RD) (RJLP) classified all marketing occasions according to their healthfulness using composite rankings (“Most Healthy”, “Less Healthy”, “Least Healthy”) (Table 1) informed by provincial nutrition guidelines [10–12]. Classifications were checked by a second RD (KDR). We calculated the repetition of food marketing in each site, defined as the number of products, brands, or retailers that were marketed at least three times per site. A FoodMATS score was derived for each site based on the ‘exposure’ to food and beverage marketing (defined as the frequency and repetition), and the ‘power’ of each marketing occasion (defined as the persuasiveness of marketing represented by its unhealthfulness, use of child-targeted and/or sports-related techniques, and size). Our definitions of exposure and power were operationalized from the WHO’s *Exposure and Power of Marketing Messages* model where exposure was explained as “the reach and frequency of the marketing message”, and power was “the creative content, design and execution of the marketing message” [13] (p.11). Scores could range from zero to infinity with higher scores representing sites with greater exposure to food marketing, along with more powerful food marketing.

**Table 1:** Classification of Marketing Occasions by Healthfulness [19]

Type	“Most Healthy”	“Less Healthy”	“Least Healthy”
Products^a^/ Brands^b^	Unprocessed foods and beverages with no added fat, sugar or salt	Foods and beverages with some added fat, sugar, or salt	Processed energy-dense, nutrient-poor items with high levels of fat, sugar, or salt
Retailers^c^	Grocery stores, farmers’ markets Sandwich outlets, smoothie outlets, salad bars	Sit-down restaurants, cafeterias, coffee outlets, prepared grocery stores, supplement stores	Pizza, burger, taco, fried chicken, Asian, and ice cream outlets, pubs, lounges, alcohol stores
Other	All nutrition education or healthy eating promotion	None	None

^a^defined as a tangible food or beverage [14]),

^b^defined as a name or symbol that represents the maker of a product [14]),

^c^defined as a place where food can be purchased (store, restaurant, etc.)

The FoodMATS was previously validated by assessing correlations with recreation facility sponsorship and advertising dollars, and whether FoodMATS scores predict unhealthy food and beverage sales [19]. During pilot testing the FoodMATS demonstrated very good to excellent inter-rater reliability (κ = 0.88–1.00, *p* < 0.001; ICC = 0.97, *p* < 0.001) [19].

Detailed methods on EPL and the FoodMATS have been previously reported [19].

We also assessed post hoc whether food marketing was related to the types of foods available for customers to purchase (as opposed to any alternative such as the food marketing was related to sponsorship or funding provided to the site by an outside organization) by identifying “in house” products, brands, and retailers. Products and brands were considered “in house” if they were sold in vending machines or concessions within the site the marketing was found. Food retailers were considered “in house” if they sold food or beverages within the site. Audits conducted at concessions and in vending machines and product sales reports collected for the EPL study were used to check whether a product or brand was sold onsite. Names of concessions recorded in the FoodMATS were used to determine if the marketed food retailer was onsite. The classification was completed by a trained graduate research assistant and checked by RJLP. This type of classification may be important to understand how food marketing is influenced across different operational areas in the facility, which may require different interventions if an association is found. For example, if most marketing is for foods and beverages available onsite then food service operators may be the target of interventions. On the other hand, if there is marketing from outside retailers or for products/brands not sold within the facility, then an intervention may need to target management or financial departments that contract out advertising space.

### Data analysis

FoodMATS data were entered, cleaned, and scored in Microsoft Excel 2013. Statistical analysis was completed wiht Statistical Package for the Social Sciences Version 23 (SPSS Inc., Chicago, IL, USA) was used with p<0.05 indicating statistical significance. Medians and interquartile ranges were used to describe the frequency and repetition of marketing, and FoodMATS scores. The prevalence of powerful features (healthfulness, child-targeted, sports-related, size) was described using proportions. Crosstabs were used to assess whether marketing occasions that used child-targeted and sports-related marketing techniques differed by healthfulness.

Differences between guideline and non-guideline provinces were assessed using Pearson’s Chi squared tests of homogeneity. Ordinal variables were collapsed into dichotomous groups to improve stability. Healthfulness was grouped into “Most Healthy”/“Less Healthy” versus “Least Healthy” as the latter are recommended to be restricted or not available in recreation facilities [10–12]. Size was grouped into small/medium versus large. Effect sizes are reported as Phi coefficients interpreted as 0.1 for small effects, 0.3 for medium effects, and 0.5 for large effects [20].

Due to unequal variances and non-normality, Mann-Whitney U tests were used to test differences between guideline and non-guideline provinces for food marketing frequency, repetition, and FoodMATS scores. Post hoc power analyses with G*Power (v3.1) revealed that our sample size would have 73% chance of detecting a large effect (D *=* 0.80, *t* = 2.01, α = 0.05) when using Mann-Whitney tests to compare mean ranks between two groups, and assuming two-tailed normal distribution with α = 0.05; but would be insufficient to detect medium (D = 0.50, α =0.36) or small (D = 0.2, α =0.099) effect sizes.

## Results

### Characteristics of guideline and non-guideline sites

The majority of guideline (*n* = 23, 67.6%) and non-guideline (*n* = 15, 88.2%) sites had one concession. Eight sites in the guideline provinces had no concession(s) (23.5%). Zero sites in the non-guideline province had no concession(s). All other sites in guideline provinces (*n* = 5, 14.7%) and the non-guideline province (*n* = 2, 11.8%) had two or more concessions. Thirty-one guideline sites (91.2%) and all 17 non-guideline sites (100.0%) had snack and/or beverage vending machines. Almost two-thirds of sites in the guideline provinces (*n* = 22, 64.7%) and non-guideline province (*n* = 11, 64.7%) had between one and four sports areas (see Table 2 for types of sports areas). One site in the guideline provinces had spaces for community events such as dances but no dedicated sport area. All other sites in the guideline (*n* = 11, 32.4%) and non-guideline provinces (*n* = 6, 35.3%) had five or more sports areas.

**Table 2:** Number and proportion of sports areas with food marketing present (*n* = 188)

	All sites		Guideline sites		Non-guideline sites	
Sports area	n	Proportion of sports areas with food marketing present (%)	n	Proportion of sports areas with food marketing present (%)	n	Proportion of sports areas with food marketing present (%)
All sports areas	188	36.2	119	34.5	69	41.2
Arenas	64	81.3	30	83.3	34	79.4
Fields	7	71.4	5	80.0	2	50.0
Tracks	4	25.0	3	66.7	1	0.0
Weight/Cardio room	24	25.0	19	31.6	5	0.0
Pool	24	12.5	16	18.8	8	0.0
Gymnasiums	38	2.6	32	3.1	6	0.0
Single-use courts	12	0.0	4	0.0	8	0.0
Cycle studios	6	0.0	5	0.0	1	0.0
Rock climbing walls	1	0.0	0	0.0	1	0.0
Other sport areas^a^	8	0.0	5	0.0	3	0.0

^a^Includes: indoor playground (*n* = 2), gymnastics area (*n* = 2), shuffle board (*n* = 1), ballet studio (*n* = 1), bowling alley (*n* = 1), skateboarding area (*n* = 1)

Food marketing was present in all but one site (*n* = 50, 98.0%), located in a guideline province. Most sites had food marketing in their food (concession) area(s) (*n* = 41 out of 43 sites with concessions, 95.3%), sports area(s) (*n* = 35 out of 50 sites with sports areas, 70.0%), and other area(s) (*n* = 46 out of 51 sites, 90.2%). Presence of food marketing differed between sport area types, ranging from 2.6% of gymnasiums to 81.3% of arenas having food marketing (Table 2). No single use courts, cycling studios, climbing areas, or other areas contained food marketing (Table 2).

### Exposure

#### Frequency

A total of 1740 food marketing occasions were recorded across all sites. The frequency of promotions by location can be found in Table 3. Overall, the median number of food marketing occasions per site was 29 (IQR 13, 42) (Table 4). There was no statistical difference between the number of food marketing occasions between provinces with and without guidelines (*p* = 0.576) (Table 4).

**Table 3:** Number and proportion of food marketing occasions found in food, sports, and other area by type (*n* = 1740)

Food (concession) areas	n	Proportion of all food marketing in food areas (%)^e^	Sports areas	n	Proportion of all food marketing in sports areas (%)^e^	Other areas	n	Proportion of all food marketing in other areas (%)^e^
Checkout	229	30.8	Playing area	200	39.3	Indoor walls/ floors	70	14.4
Price promotions^a^	159	21.3	Seating area	96	18.9	Facility TVs	24	4.9
Signs/ displays/ table tents	150	20.2	Other^c^	59	11.6	Other^d^	22	4.5
Menus	102	13.7	Scoreboard/clocks	44	8.6	Outdoor walls, windows, doors	14	2.9
Other^b^	101	13.6	Change/locker rooms	15	2.9	Welcome desk	14	2.9
						Outdoor signs, furniture	10	2.1
						Facility pamphlets	10	2.1
						Bathrooms	3	0.6
Vending machines	3	0.4	Vending machines in spectator area	61	12.0	Vending machines	320	65.7
			Vending machines in athlete area	34	6.7			
Total	744	100.0	Total	509	100.0	Total	487	100

^a^Includes multiple pricing promotion types: combos; small versus regular portions; and healthy entrees, salads, beverages, and snacks versus regular; and other pricing. No supersize, all-you-can-eat, free refills, loyalty programs were found

^b^Includes marketing/branding on fridges, coolers, machines, garbage cans, recycling cans, menus, clocks etc.

^c^Includes marketing/branding on stairs, coolers, floors, bulletin boards, etc.

^d^Includes marketing on sandwich boards/posters

^e^Percentages may not add up to 100.0 due to rounding

**Table 4:** Exposure to food and beverage marketing occasions for facility areas for guideline and non-guideline provinces (*n* = 1740)

	All sites (*n* = 51)		Guideline sites (*n* = 34)		Non-guideline sites (*n* = 17)		
	Median	IQR^a^	Median	IQR^a^	Median	IQR^a^	*P* value^b^
Frequency of food marketing occasions (n)							
Total Site	29.0	13.0, 42.0	28.5	5.5, 42.3	29.0	20.0, 42.5	*p* = 0.576
Food Areas	13.0	7.3, 20.8	15.0	5.0, 25.0	12.0	7.5, 17.0	*p* = 0.447
Sports Areas	5.5	0.0, 13.0	6.0	0.0, 15.0	5.0	2.0, 12.5	*p* = 0.787
Other Areas	7.0	3.0, 13.0	7.0	3.0, 13.0	11.0	3.5, 15.5	*p* = 0.389
Repetition of food marketing occasions (n)							
Total Site	2.0	1.0, 3.0	2.0	1.0, 3.0	2.0	1.0, 3.0	*p* = 0.217

^a^Interquartile Range (IQR) = 25th percentile, 75th percentile

^b^asymptotic significance (2-tailed) from Mann-Whitney test difference of mean ranks between scores

Products or brands were most frequently marketed, comprising 75.3% of all marketing occasions. The remaining food marketing occasions promoted food retailers (22.5%) or were nutrition education or general healthy eating promotions (2.2%), such as government, industry, or site developed posters that provided nutrition information or highlighted healthy food choices. Most products (97.1%) and brands (85.8%) marketed were “in house”, but only 12.7% of marketing occasions for food retailers were “in house”. Food retailers that did not sell food within the facility were promoted almost eight times more often than “in house” food retailers.

#### Repetition

Overall, sites marketed a median of two products, brands, or retailers three or more times. However, the top quartile of sites repeatedly marketed between three and 13 products, brands, and retailers at least three times within their site. There was no difference in the number of repeated products, brands, and retailers between guideline and non-guideline provinces (*p* = 0.217) (Table 5).

**Table 5:** Power of food and beverage marketing occasions for guideline and non-guideline provinces (*n* = 1740)

Power feature	All sites (*n* = 51)		Guideline sites (*n* = 34)		Non-guideline sites (*n* = 17)		
	n (missing)	%	n (missing)	%	n (missing)	%	*P* value^a^
Healthfulness	*n* = 1740 (0)		*n* = 1212 (0)		*n* = 528 (0)		*p* < 0.001
Most Healthy	420	24.1	358	29.5	62	11.7	
Less Healthy	352	20.2	274	22.6	78	14.8	
Least Healthy	968	55.6	580	47.9	388	73.5	
Child-targeted^b^	*n* = 1377 (5)		*n* = 953 (4)		*n* = 424 (1)		*p* < 0.001
Targeted at children	99	7.2	91	9.5	8	1.9	
Sports-related^c^	*n* = 1377 (5)		*n* = 953 (4)		*n* = 424 (1)		*p* < 0.001
Related to sports	123	8.9	104	10.9	19	4.5	
Size total	*n* = 1375 (6)		*n* = 952 (4)		*n* = 423 (2)		*p* = 0.001
Small^d^	444	32.3	282	29.6	162	38.3	
Medium^e^	257	18.7	193	20.3	64	15.1	
Large^f^	674	49.0	477	50.1	197	46.6	

^a^asymptotic significance (2-sided) from Chi^2^ tests for homogeneity

^b^evidence of animated or fictional characters, taste appeals, humour, action-adventure, fantasy, fun shapes or colours, competitions, give-aways, cartoonish font, or used a child actor to advertise a food or beverage product/brand that would appeal to children (Prowse et al. submitted to *IJBNPA* November 2017, IJBN-D-17-00585)

^c^any reference to physical activity, exercise, sport, game, recreation, performance or competition, a design feature relevant to sport settings (Prowse et al. submitted to *IJBNPA* November 2017, IJBN-D-17-00585)

^d^small: less than one 8.5 × 11 in. paper (Prowse et al. submitted to *IJBNPA* November 2017, IJBN-D-17-00585)

^e^outdoor medium: one to ten 8.5 × 11 in. paper(s); indoor medium: one to three 8.5 × 11 in. paper(s) (Prowse et al. submitted to *IJBNPA* November 2017, IJBN-D-17-00585)

^f^outdoor large: more than ten 8.5 × 11 in. paper(s); indoor large: more than three- 8.5 × 11 in. paper(s) (Prowse et al. submitted to *IJBNPA* November 2017, IJBN-D-17-00585)

### Power

There were statistically significant differences in the proportions of food marketing occasions that were “Least Healthy”, child-targeted, sports-related, and large size between sites in guideline and non-guideline provinces (Table 5).

#### Healthfulness of marketing

Overall, more than half of all food marketing occasions were considered “Least Healthy” (55.6%) (Table 5). There was a significantly greater proportion of “Least Healthy” food marketing occasions in the non-guideline province compared to the guideline province (X^2^ (1, *N* = 1740) =63.604, Phi coefficient = − 0.191, *p* < 0.001) (Table 5).

#### Child-targeted food marketing

Approximately, one in every 14 food marketing occasions (7.2%) was targeted at children (Table 5). There was a significantly greater proportion of child-targeted food marketing occasions in guideline provinces than in non-guideline provinces (X^2^ (1, *N* = 1377) =25.817, Phi coefficient = 0.137, *p* < 0.001) (Table 5).

**Fig. 1:**
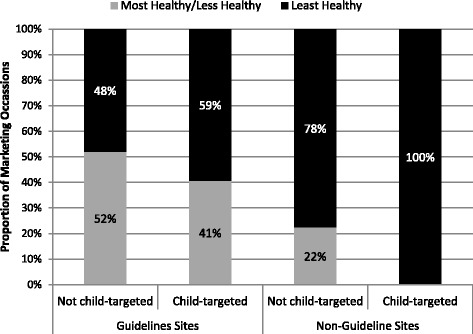
Distribution by healthfulness for child-targeted and non-child-targeted marketing occasions in guideline and non-guideline provinces (*N* = 1377)

Across all sites, the healthfulness of food marketing occasions targeted at children and not targeted at children were similar, however, 100.0% of the food marketing occasions targeted at children in non-guideline provinces were “Least Healthy” (*n* = 8), compared to only 59.3% in guideline provinces (*n* = 54) (Fig. 1).

#### Sports-related food marketing

Approximately 1 in every 11 food marketing occasions (8.9%) were sports-related (Table 5). There was a significantly greater proportion of sports-related food marketing occasions in guideline provinces than in the non-guideline province (X^2^ (1, *N* = 1377) =14.923, *p* < 0.001, Phi coefficient = 0.086) (Table 5).

**Fig. 2:**
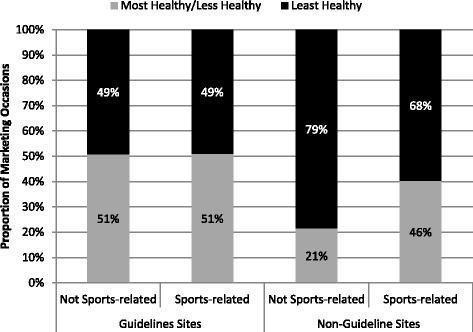
Distribution by healthfulness for sports-related and non-sports-related marketing occasions in guideline and non-guideline provinces (*N* = 1377)

Overall, 52.0% of all sports-related food marketing occasions were “Least Healthy” (*n* = 64); however, it was more common in non-guideline sites with 68.4% (*n* = 51) to have sports-related food marketing occasions for “Least Healthy” products, brands, or retailers compared to 49.0% (*n* = 53) in guideline sites (Fig. 2).

#### Size of marketing

Almost half of all food marketing occasions were large and one-third were small (Table 5). There was a significantly greater proportion of large food marketing occasions in the guideline province than the non-guideline provinces (X^2^ (2, *N* = 1375) =11.718, Phi coefficient = 0.092, *p* = 0.003) (Table 5).

### FoodMATS scores

Overall, the median score was 43.3 (IQR 18.6, 71.0) with higher scores indicating greater exposure to food marketing, along with more powerfulfood marketing. There was no statistically significant difference in FoodMATS scores between guideline (median = 42.7, IQR 4.6, 70.1) and non-guideline provinces (median = 43.3, IQR 29.5, 71.5).

## Discussion

Food marketing, such as signs, posters, branding, pricing promotions, and product placement, was found to be present in almost all recreation facility sites with unhealthy products, brands, or retailers marketed on more than half of the occasions. Our study found mixed results in differences between sites in guideline and non-guideline provinces, differing by ‘power’ (healthfulness of food marketing, targeting children, using appeals of physical activity, and having large signs) but not by ‘exposure’ (frequency, and repetition) nor FoodMATS scores (the composite of ‘power’ and ‘exposure’).

It may be surprising that the FoodMATS scores did not differ between guideline types despite differences in ‘power’. This null result may be related to the fact that we could only use a non-parametric test to compare mean ranks. If actual values were assessed, findings may have shown a difference since the 25th percentile of FoodMATS score is almost 25 points (84.4%) lower in the guideline provinces than in the non-guideline province. Secondly, FoodMATS scores were calculated by assessing each component of ‘power’ individually rather than cumulatively. If ‘power’ was scored based on the cumulative presence of marketing techniques, the FoodMATS scores in the non-guideline province may have been higher since more marketing occasions that used child-targeted and sports-related techniques were for “Least Healthy” products, making it easier to see differences between guideline types. However, our approach of evaluating each component individually proposes the idea that the impact of food marketing on children’s food preferences and behaviours may remain unchanged if one marketing technique is replaced by another (e.g. replace sports-related food marketing occasions with child-targeted food marketing occasions).

The lack of difference in FoodMATS scores may highlight that there are multiple components to food marketing that need to be considered in policy interventions. Current provincial nutrition guidelines incompletely address food marketing by merely recommending what product should or should not marketed (i.e. healthy versus unhealthy food) which is only one component of marketing strategies. How foods and beverages are marketed (targeted to children, sports-related, and physical size, as well as potentially other characteristics not assessed in this study) should also be regulated in order to protect children from exposure to powerful food marketing. That being said, protecting children’s environments from all unhealthy food marketing would reduce children’s exposure to food marketing and thus make discussions regarding other powerful features redundant.

No previous research has evaluated food marketing in sports settings as comprehensively as this study. Carter et al. [21] identified 131 food and beverage companies that advertised on sports clubs’ websites in New Zealand. Although we did not measure the number of different marketers, we found that only a couple products, brands, and/or retailers were marketed repeatedly in a site. The findings from both Carter et al. [21] and this study suggest that there are several food industry actors involved in food marketing in recreation and sport facilities. Kelly et al. [22] found that sports club food sponsors in Australia most commonly provided jersey branding (53% of sponsors), official partnership (52%), recognition in club newsletters (29%), signs (28%), and onsite availability of sponsors’ product (24%). This project also found that signage was a common marketing channel and that most products marketed were available for purchase in the facility. However, the marketing techniques and channels captured by Kelly et al. [22] only overlap to a limited extent with the FoodMATS since Kelly et al. [22] only evaluated sponsorship and the FoodMATS broadly assessed food marketing within multiple areas of the facility including concessions and vending machines. The breadth of food marketing found in this study suggests that sponsorship may be only one of many strategies the food industry uses to market their product, brand, or retailer in sports settings.

The proportion of marketing occasions that were “Least Healthy” (55%) found in this study is similar to the proportion of food sponsors classified as unhealthy by Carter et al. [21] (using the New Zealand Food and Beverage Classification System) and Kelly et al. [22] (through expert consensus classification). These consistent findings suggest that food marketing environments in recreation and sport facilities are not health promoting.

The greater use of child-targeted marketing in the guideline province may reflect that the provincial guidelines tend to focus on improving children’s environments and may be related to efforts by sites from guideline provinces to move towards offering and promoting healthier options for children. It could also be explained by other factors that we did not assess including differences in the prevalence of onsite child programming or proximity of schools to the recreation facility.

The difference in sports-related marketing between guideline and non-guideline provinces is surprising because the prevalence of sports areas with food marketing was lower in the guideline provinces than in the non-guideline province, and the number of sports areas was similarly distributed in both groups. The study did find that food marketing was variable depending on the type of sport, consistent with previous research [21, 23]. Despite this, it is unclear whether differences in sport types between sites in guideline and non-guideline provinces explains the different prevalence of sports-related food marketing between guideline types.

### Strengths and limitations

The results of this study must be interpreted cautiously due to its cross-sectional design and small, non-representative sample; yet, this is the largest known assessment of food marketing in recreation facilities in Canada. Unfortunately, our small sample size did not allow us to investigate whether differences in marketing environments existed in sites between guideline provinces in relation to their variable food marketing recommendations. Similarly, we had insufficient power to adjust for clustering effects within provinces resulting in confidence intervals narrower than if we could have adjusted for clustering. Despite its limitations, the FoodMATS is a theoretically grounded reliable validated tool that provides broad and detailed information on food marketing. Although it did not measure sponsorship specifically, it captured a breadth of marketing approaches the food industry uses in sport settings.

### Implications & recommendations

To our knowledge, this is the first study to investigate the ‘exposure’ and ‘power’ of food marketing in sport settings, a place where children gather that should be free from unhealthy food marketing [18], and to examine differences in food marketing environments according to presence of regional voluntary nutrition guidelines. We found differences between what and how foods and beverages were marketed, but not in the frequency or repetition of marketing. Findings suggest that the presence of voluntary provincial nutrition guidelines that focus on what food provision rather than food marketing may be insufficient to impact the frequency of marketing but may influence the healthfulness of marketing. It is possible that provincial nutrition guidelines improve the foods available for sale onsite which impacts their marketing. However, nutrition guidelines for food provision can only be expected to go so far; a study of food promotions in public schools in Vancouver, Canada found that almost one-quarter of promotions were for “Choose Least” and “Not Recommended” foods and beverages [24] even though provincial school nutrition guidelines there discouraged unhealthy food marketing (e.g. posters, coupons, and branded equipment) [25].

The presence of unhealthy food marketing found in schools by Velazquez et al. [24] and in recreation facilities presented here despite the presence of nutrition guidelines suggests that it should not be assumed that healthy food provision policies will translate to healthier food promotion. On the other hand, it may also be shortsighted to assume that food provision policies will have no impact on food marketing within its applicable setting.

Although child-targeted marketing techniques were used infrequently, recreation and sport facilities still offer multiple exposures to unhealthy food marketing. Regardless of their power, children will likely still see such marketing and be impacted by it. Sport sponsorship is not inherently child-targeted, but a study of 5–12 year olds in New Zealand found that 76% of children can correctly match sponsors to their respective sport [26]. Pettigrew et al. [26] also found that even when children mismatched sponsors with sports, 83% of children selected an unhealthy food brand for that sport, suggesting that children have a strong association of unhealthy food with sport. A photo-based project in New Zealand revealed that 83% of beverages 10–12 year olds associate with sport were not consistent with dietary guidelines [27].

The presence of unhealthy food marketing in almost all recreation facilities studied in Canada is worrisome from a population health perspective. Thousands of children, youth, and families use public recreation facilities in Canada [28, 29], thus the reach of food marketing is broad. Kelly et al. [23] estimated that Australian children may be exposed up to 64,000 person-hours of food and beverage sponsorships per week depending on the sport. It is not reasonable to expect recreation facilities that sell food to be free of food marketing (although food sponsorship may be unnecessary), but marketing environments could be improved to be less pervasive across recreation and sport facilities and be used to promote healthy products only. Marketing policies that reduce ‘exposure’ to and ‘power’ of food and beverage marketing are recommended by the WHO [18] and could reduce the impact of unhealthy food marketing on children’s eating behaviors. Institutions, such as recreation facilities, may consider generating food marketing restrictions to complement food provision policies s in order to more comprehensively promote healthy diets [30].

Future research should explore the relationships of food marketing in children’s sport settings with other environmental factors (food availability, food sales) and the impact of food marketing in sport settings on individual and population diet and health outcomes. Investigating the impact of food marketing according to FoodMATS scores may help to understand how to reduce the impact of food marketing by identifying ideal food marketing scores and generating strong, specific recommendations for policymakers to restrict unhealthy food marketing and sponsorship in children’s sport settings. Researchers should consider assessing differences in food marketing between sport types (hockey versus soccer), facility type (public versus private funding; single versus multi-sport), competition levels, and communities in which these facilities are located (high versus low income; urban versus rural).Such research may reveal whether certain populations are at greater risk of exposure to unhealthy food marketing environments. Understanding such differences could identify where to focus interventions to have the greatest population impact on diet, health, and childhood obesity.

## Conclusions

It is argued that the food industry often overemphasizes the importance of physical activity deliberately [31, 32] to “[deflect] attention from its possible role in the obesity epidemic” (p.244) [33]. The overwhelming presence of food marketing in recreation facilities may be evidence of one method used by the food industry to do so. Over half of food products, brands, and retailers marketed in public recreation facilities were “Least Healthy”. Although not common, child-targeted and sports-related features were occasionally present. Having provincial nutrition guidelines did not appear to impact the frequency or repetition of food marketing in recreation facilities, but was associated with less unhealthy food promotion, including the products marketed with child-targeted or sports-related techniques. As researchers and practitioners work to improve food environments in sport settings, targeting food marketing as an environmental factor appears important for supporting healthy eating.
